# Inhibition of mTOR pathway by everolimus cooperates with EGFR inhibitors in human tumours sensitive and resistant to anti-EGFR drugs

**DOI:** 10.1038/sj.bjc.6604269

**Published:** 2008-03-04

**Authors:** R Bianco, S Garofalo, R Rosa, V Damiano, T Gelardi, G Daniele, R Marciano, F Ciardiello, G Tortora

**Affiliations:** 1 Cattedra di Oncologia Medica, Dipartimento Endocrinologia e Oncologia Molecolare e Clinica, Università di Napoli Federico II, via S. Pansini 5, Napoli 80131, Italy; 2 Dipartimento Medico-Chirurgico di Internistica Clinica e Sperimentale, Seconda Università di Napoli, via S. Pansini 5, Napoli 80131, Italy

**Keywords:** angiogenesis, drug resistance, EGFR, everolimus, mTOR

## Abstract

Inhibition of a single transduction pathway is often inefficient due to activation of alternative signalling. The mammalian target of rapamycin (mTOR) is a key intracellular kinase integrating proliferation, survival and angiogenic pathways and has been implicated in the resistance to EGFR inhibitors. Thus, mTOR blockade is pursued to interfere at multiple levels with tumour growth. We used everolimus (RAD001) to inhibit mTOR, alone or in combination with anti-EGFR drugs gefitinib or cetuximab, on human cancer cell lines sensitive and resistant to EGFR inhibitors, both *in vitro* and *in vivo*. We demonstrated that everolimus is active against EGFR-resistant cancer cell lines and partially restores the ability of EGFR inhibitors to inhibit growth and survival. Everolimus reduces the expression of EGFR-related signalling effectors and VEGF production, inhibiting proliferation and capillary tube formation of endothelial cells, both alone and in combination with gefitinib. Finally, combination of everolimus and gefitinib inhibits growth of GEO and GEO-GR (gefitinib resistant) colon cancer xenografts, activation of signalling proteins and VEGF secretion. Targeting mTOR pathway with everolimus overcomes resistance to EGFR inhibitors and produces a cooperative effect with EGFR inhibitors, providing a valid therapeutic strategy to be tested in a clinical setting.

The mammalian target of rapamycin (mTOR), also known as FKBP12 rapamycin-associated protein, rapamycin target or sirolimus effector protein, is a serine/threonine-specific kinase responsible for mitogen-induced cell proliferation/survival signalling (Bjornsti and Houghton, 2004; Abraham and Gibbons, 2007). In mammalian cells, mTOR signalling depends on signal transmission through the phosphoinositide 3-kinase (PI3K)/Akt pathway. Activated Akt phosphorylates TSC2, a component of tuberous sclerosis complexes 1 and 2, sensitive to nutrient status, and favours mTOR activity (Manning *et al*, 2002; Zhang *et al*, 2003). An important function of mTOR is the translation control via activation of ribosomal p70S6 kinase (S6K1) and suppression of 4E-BPs, resulting in enhanced translation of mRNAs encoding cell-cycle regulators and promotion of G1–S cell-cycle transition (Schmelzle and Hall, 2000; Bjornsti and Houghton, 2004). The mammalian target of rapamycin signalling is dependent on insulin, growth factors, and nutrition status, and nutrient deficiency leads to mTOR inhibition.

Mammalian target of rapamycin is an essential part of tumour growth being capable of integrating proliferative, antiapoptotic and angiogenic signalling by connecting VEGF, hypoxia-inducible factor 1 (HIF-1) and HER family receptors. VEGF is a potent promoter of angiogenesis, and its overexpression is associated with tumour progression and poor clinical outcome. Hypoxia-inducible factor 1, a heterodimeric protein consisting of HIF-1*α* and HIF-1*β*, controls the transcription of genes involved in cell proliferation/survival, invasion/metastasis and angiogenesis, including VEGF. Hypoxia-inducible factor 1 is degraded through ubiquitylation involving the von Hippel–Lindau tumour-suppressor protein, whose loss of function, detected in renal cancer and neuroendocrine tumours, impairs HIF-1*α* degradation and favours the stimulation of mTOR signalling (Melillo, 2007). Finally, it has been reported that HER2 activation in normoxic cells increases HIF-1*α* via PI3K, Akt and mTOR (Laughner *et al*, 2001), and that EGF induces HIF-1*α*, which can be prevented by using mTOR inhibitors under both normoxic and hypoxic conditions (Phillips *et al*, 2005). Taken together, these studies demonstrate that mTOR is a positive regulator of HIF-1*α* translation and an inducer of HIF-1/VEGF-dependent angiogenesis. In addition, signalling through mTOR is stimulated by defects in the pathway components upstream of mTOR, such as growth factor receptors, PI3K, Akt, PTEN, TSC1/TSC2, or by stimulation of PI3K by effectors of the mutant Ras/Raf/MAPK pathway (Bjornsti and Houghton, 2004).

In view of the above-mentioned facts, several mTOR inhibitors rapamycin-analogues have been developed, including temsirolimus (CCI-779), everolimus (RAD001) and AP23573 (Hidalgo and Rowinsky, 2000; Rowinsky, 2004). Clinical studies have been very encouraging in renal cell cancer. In a multicenter phase 3 trial, temsirolimus improved overall survival in metastatic renal-cell carcinoma patients with poor prognostic score, as compared with IFN-*α*; however, the addition of temsirolimus to IFN did not improve survival (Hudes *et al*, 2007). The potential efficacy of these new agents is now under evaluation in patients with other solid tumours (Janus *et al*, 2005; Smolewski, 2006; Fouladi *et al*, 2007).

Everolimus (40-*O*-(2-hydroxyethyl)-rapamycin) is an orally bioavailable derivative of rapamycin under clinical evaluation in different types of cancer (Boulay *et al*, 2004), including renal cell cancer and endometrial cancer (Sansal and Sellers, 2004). Moreover, everolimus in combination with imatinib mesylate is under study in patients with gastrointestinal stromal tumours (Van Oosterom *et al*, 2004). Phase I/II trials of everolimus in combination with erlotinib, in patients with metastatic breast cancer and advanced NSCLC are currently undergoing.

EGFR is a major transducer of mitogenic signals involved in cancer pathogenesis and progression (Citri and Yarden, 2006) upstream of mTOR and an important target for anticancer therapy (Baselga and Arteaga, 2005; Hynes and Lane, 2005; Mendelsohn and Baselga, 2006). Several anti-EGFR drugs, such as monoclonal antibodies directed against the extracellular domain and small molecule inhibitors of the receptor-associated tyrosine kinase, have been approved for clinical use (Cunningham *et al*, 2004; Bezjak *et al*, 2006). However, a relevant percentage of patients treated with these drugs has no clinical benefit or incurs in the development of acquired resistance. Constitutive and acquired resistance to EGFR inhibitors in a large number of patients is a relevant clinical problem. Frequently, activation of signalling pathways downstream or alternative to EGFR may be responsible for the development of resistance to these inhibitors (Sansal and Sellers, 2004). We, and others, have previously shown that colon tumours that acquire resistance to anti-EGFR drugs cetuximab and gefitinib overexpress Akt and VEGF, which act as the escape pathways to overcome the EGFR blockade (Viloria-Petit *et al*, 2001; Ciardiello *et al*, 2004; Bianco *et al*, 2005). As mentioned above, the PI3K-AKT pathway connects EGFR and mTOR.

The link between HIF-1 and EGF and the reported ability of mTOR inhibitors rapamycin and everolimus to reduce VEGF secretion *in vitro* and *in vivo*, renders mTOR inhibitors potentially effective against cancer cells resistant to EGFR inhibitors (Viloria-Petit *et al*, 2001; Ciardiello *et al*, 2004; Bianco *et al*, 2005). In addition, targeting together mTOR and EGFR might cause a more profound effect on tumour growth control compared with a single agent (Hynes and Lane, 2005).

In this study, we have evaluated (1) the antitumour effect of everolimus on different human cancer cell lines, sensitive or resistant to EGFR inhibitors, *in vitro* and in nude mice; (2) the possibility to restore sensitivity to EGFR inhibitors, using everolimus in combination with gefitinib or cetuximab; (3) the effect of treatment on signalling pathways and VEGF.

## MATERIALS AND METHODS

### Drugs

Everolimus, gefitinib and cetuximab were provided by Novartis International AG (Basel, Switzerland), Dr Anderson Ryan (AstraZeneca Pharmaceuticals, Macclesfield, UK) and ImClone Systems (New York, NY, USA).

### Cell lines

Human GEO colon, PC3 prostate and MDA-MB-468 breast cancer cells were obtained from the American Type Culture Collection (Manassas, VA, USA). GEO-CR (cetuximab resistant), GEO-GR and PC3-GR (gefitinib resistant) cells were established as described previously (Ciardiello *et al*, 2004).

### Growth in soft agar

Cells (10^4^ cells per well) were suspended in 0.3% Difco Noble agar (Difco, Detroit, MI, USA) in culture medium, layered over 0.8% agar medium base layer in 24-well plates (Becton Dickinson, Lincoln Park, NJ, USA) and treated with gefitinib, cetuximab or everolimus. After 10–14 days, cells were stained and colonies up to 0.05 mm were counted (Ciardiello *et al*, 2004).

### Cell-survival assay

Cells were grown in 24-well plates in the presence of everolimus (1 nM to 1 *μ*M), gefitinib (1 or 5 *μ*M) or their combination. After removing supernatant, 1 mg ml^−1^ of 3-(4,5-dimethylthiazol-2-yl)-2,5-diphenyltetrazolium bromide (MTT, Sigma, Milan, Italy) solution in medium was added to each well. After adding isopropanol, absorbance was measured at 570 nm.

### Western blot analysis

Total cell lysates were obtained from homogenised tumour specimens or from cells. Protein extracts resolved by a 4–20% SDS-PAGE were probed with different antibodies: anti-Akt, anti-phospho (Ser473)-Akt (Cell Signaling, Beverly, MA, USA); anti-ERK 1/2, anti-phospho-ERK 1/2 (Santa Cruz Biotechnology, Santa Cruz, CA, USA); anti-S6K1, anti-phospho S6K1, anti-VEGF (Santa Cruz Biotechnology); anti-actin (Sigma-Aldrich, Milan, Italy). Immunoreactive proteins were visualised by enhanced chemiluminescence (Amersham International, London, UK) (Ciardiello *et al*, 2004).

### ELISA assay

VEGF concentrations in conditioned media, total cell lysates from homogenised tumour specimens and serum of sacrificed mice were determined by ELISA (Guba *et al*, 2002). The absorbance was measured at 490 nm on a microplate reader (Bio-Rad, Hercules, CA, USA) and VEGF concentrations were determined using linear regression analysis.

### Xenografts in nude mice

Balb/cAnNCrlBR athymic (nu+/nu+) mice (Charles River Laboratories, Milan, Italy) were maintained in accordance with institutional guidelines of the University of Naples Animal Care Committee and Declaration of Helsinki. GEO and GEO-GR cells (10^7^ cells per mice) resuspended in Matrigel (Collaborative Biomedical Products, Bedford, MA, USA) were injected subcutaneously. After 7 days, groups of 10 mice were randomised to receive treatments: intraperitoneal gefitinib, 100 mg kg^−1^, three times a week for 3 weeks; everolimus (by gavage), 5 mg kg^−1^, three times a week for 3 weeks; or their combination, on days 7–11, 14–18 and 21–25. Tumour volume was measured using the formula *π*/6 × larger diameter × (smaller diameter)^2^ (Ciardiello *et al*, 2004). Two mice were sacrificed on day 25 for biochemical analysis.

### Vascular endothelial cell capillary tube and network formation

Matrigel was diluted in DMEM, added into a 30-mm culture dish and incubated at 37°C for 30 min; then HUVECs (4 × 10^5^) in RPMI medium were added in each dish, in presence of everolimus 0.1 *μ*M, gefitinib 5 *μ*M or the combination. The positive control was matrigel with VEGF 100 ng ml^−1^ (R&D Systems, Minneapolis, MN, USA). Pictures were taken at 0 and 24 h.

### Statistical analysis

The Student's *t*-test and the Mantel–Cox log-rank test were used to evaluate the statistical significance of the results. All *P*-values were two-sided. Analyses were performed with the BMDP New System statistical package version 1.0 for Microsoft Windows (BMDP Statistical Software, Los Angeles, CA, USA).

## RESULTS

### Everolimus is active in cancer cell lines resistant to gefitinib and cetuximab and partially restores sensitivity to these EGFR inhibitors

To evaluate the effect of everolimus on cancer cell growth, we have performed a soft agar assay on different cancer cells. Wild-type human colon cancer GEO cells are sensitive to both cetuximab and gefitinib, with an IC_50_ of less than 0.5 *μ*g ml^−1^ and 0.5 *μ*M, respectively. Wild-type human prostate cancer PC3 cells demonstrate the same pattern of sensitivity to gefitinib, but are resistant to cetuximab at doses up to 20 *μ*g ml^−1^. GEO-CR, GEO-GR and PC3-GR human cancer cell lines were established *in vitro* from GEO or PC3 tumours treated continuously for 16 weeks with either gefitinib or cetuximab. These derivative cell lines are resistant to cetuximab and gefitinib up to doses of 80 *μ*g ml^−1^ and 20 *μ*M, respectively (data not shown). MDA-468 human breast cancer cells exhibit low sensitivity to gefitinib (Bianco *et al*, 2003) and resistance to cetuximab up to the dose of 80 *μ*g ml^−1^ (data not shown). This constitutive resistant phenotype is associated with PI3-K/Akt hyperactivity, in turn related to mutation of the phosphatase and tensin homologue (PTEN) gene (Bianco *et al*, 2003). Despite similar sensitivity to gefitinib, GEO cells have a functional wild-type PTEN gene, whereas PC3 have a deleted PTEN. However, western blot analysis of GEO-CR, GEO-GR and PC3-GR cells did not reveal any differences in PTEN expression (data not shown).

**Figure 1:**
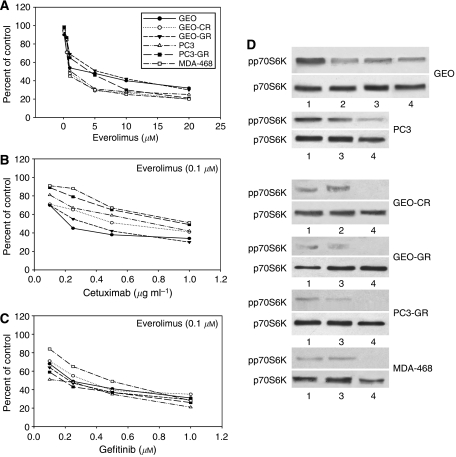
(**A**) Cells were treated with everolimus (0.01–20 *μ*M). (**B** and **C**) Cells were treated with cetuximab (0.1–1 *μ*g ml^−1^), and gefitinib (0.1–1 *μ*M), in presence of everolimus, 0.1 *μ*M. (**D**) Western blotting on total cell lysates from cells treated with 1 *μ*M gefitinib, 1 *μ*g ml^−1^ cetuximab or 0.1 *μ*M everolimus. Lane 1: untreated control; lane 2: cetuximab; lane 3: gefitinib; lane 4: everolimus.

Regardless of the degree of sensitivity to EGFR inhibitors and PTEN status, everolimus caused an efficient dose-dependent inhibition of soft agar growth in all cell lines, with an IC_50_ ranging between 1 and 5 *μ*M, (Figure 1A). To evaluate the everolimus potential to restore sensitivity to gefitinib and cetuximab in cancer cell lines resistant to these anti-EGFR drugs, we performed a soft agar assay on cells treated with doses of cetuximab or gefitinib ranging from 0.1 to 1 *μ*g ml^−1^ and from 0.1 to 1 *μ*M, respectively, in presence of everolimus 0.1 *μ*M. Wild-type GEO cells showed a reduction of cetuximab IC_50_ from 0.5 to 0.2 *μ*g ml^−1^ and gefitinib IC_50_ from 0.5 to 0.2 *μ*M. Wild-type PC3 cells showed a reduction of gefitinib IC_50_ from 0.5 to 0.2 *μ*M, and they became sensitive to cetuximab (IC_50_ 0.8 *μ*g ml^−1^). In all the resistant cell lines, everolimus in combination with cetuximab or with gefitinib restored dose-dependent growth inhibition induced by anti-EGFR drugs (Figure 1B and C).

### Everolimus inhibits mTOR pathway in resistant cancer cells

As mTOR signalling is dependent on growth factors, including EGF, we compared everolimus with gefitinib or cetuximab in both wild type and EGFR inhibitor-resistant cancer cell lines, focusing on the mTOR effector p70S6 Kinase. Western Blot analysis revealed that in wild-type GEO and PC3 cells, everolimus (0.1 *μ*M) reduces p70S6K phosphorylation similarly to gefitinib or cetuximab, confirming the role of EGFR in mTOR signalling regulation. In resistant GEO-CR, GEO-GR, PC3-GR and MDA-468 cells, EGFR inhibitors were ineffective on p70S6K activity, whereas everolimus caused a complete suppression of p70S6K phosphorylation (Figure 1D).

### Everolimus restores the survival-inhibitory effects of anti-EGFR drugs in resistant cancer cells

**Figure 2:**
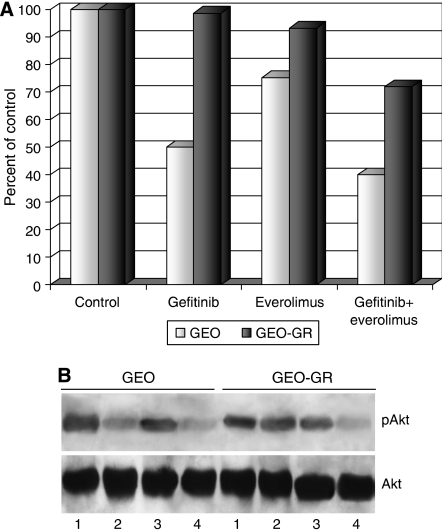
(**A**) GEO and GEO-GR cells were treated with everolimus (0.1 *μ*M) or gefitinib (5 *μ*M) alone or in combination. The results are statistically significant for everolimus plus gefitinib *vs* control, *vs* everolimus alone and *vs* gefitinib alone (two-sided *P*<0.0001). For GEO cells, the results are statistically significant for both gefitinib and everolimus *vs* control (two-sided *P*<0.0001), whereas for GEO-GR cells only for everolimus *vs* control (two-sided *P*<0.0001). (**B**) Western blotting on total cell lysates from GEO and GEO-GR cells treated with 1 *μ*M gefitinib and/or 0.1 *μ*M everolimus. Lane 1: untreated control; lane 2: gefitinib; lane 3: everolimus; lane 4: gefitinib plus everolimus.

To verify the effects of everolimus alone or in combination with gefitinib on wild-type GEO and GEO-GR cell lines, we performed a cell-survival assay. As expected, gefitinib (5 *μ*M) induced a 50% cell-survival inhibition in GEO cells, whereas it was totally ineffective on GEO-GR cells. Everolimus (0.1 *μ*M) was effective on GEO cells, with a cell-survival inhibition of about 25%, and it showed a slight activity on GEO-GR cells. Combination of everolimus and gefitinib improved the cell-survival inhibition caused by gefitinib alone in sensitive GEO cells, and it partially reverted the resistance to gefitinib in GEO-GR cells with a cell-survival inhibition of about 30% (Figure 2A). These results refer to the simultaneous administration of the two agents. This modality was chosen because previously we performed a series of experiments to test different sequences of administration of the two drugs, comparing either the use of everolimus 1, 6 or 24 h before gefitinib, or gefitinib before everolimus or their simultaneous treatment. We observed no significant differences among the different modalities used (data not shown).

As Akt is involved in pathways regulating cell survival, we investigated the effect of the combined treatment with everolimus and gefitinib on Akt phosphorylation/activation. As expected, gefitinib is able to inhibit Akt activation in GEO, but not in GEO-GR cells, whereas everolimus has no effect on Akt phosphorylation in both cell lines (Figure 2B). Conversely, the combination of everolimus and gefitinib strongly reduces pAkt levels, to a greater extent in GEO-GR than in GEO cells (Figure 2B).

### Everolimus reduces the secretion of VEGF in wild type and resistant cancer cells

**Figure 3:**
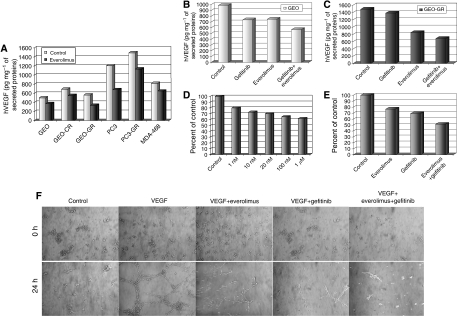
ELISA assays for hVEGF on conditioned media from (**A**) cancer cells treated with everolimus (0.1 *μ*M) and from (**B**) GEO and (**C**) GEO-GR cancer cells treated with everolimus (0.1 *μ*M) and gefitinib (5 *μ*M) alone or in combination. HUVEC cells were treated with everolimus from 1 nM to 1 *μ*M (**D**) or with everolimus 0.01 *μ*M and gefitinib 1 *μ*M alone or the combination (**E**). HUVECs were incubated on solidified matrigel in presence of everolimus 0.1 *μ*M, gefitinib 5 *μ*M or the combination. The positive control was matrigel with VEGF 100 ng ml^−1^. Photographies were performed at 0 and 24 h (**F**). All the results are statistically significant for each treatment *vs* control (two-sided *P*<0.0001).

Based on the reported ability of mTOR to affect VEGF levels (Guba *et al*, 2002), we evaluated the effect of everolimus on VEGF production in our cancer cell lines. As shown in Figure 3A, everolimus reduces human VEGF (hVEGF) secretion of 20–45% both in wild type and in resistant cell lines, compared with untreated controls. We then measured hVEGF by ELISA assay on conditioned media derived from GEO and GEO-GR cancer cell culture treated for 24 h with everolimus (0.1 *μ*M) and gefitinib (5 *μ*M) alone or in combination. Gefitinib significantly reduces hVEGF levels only in wild-type GEO cells, whereas everolimus is active in both cell lines, especially in resistant GEO-GR cells. Combined treatment with gefitinib and everolimus causes a strong reduction of hVEGF levels both in GEO and in GEO-GR cells (Figure 3B and C).

### Everolimus inhibits vascular endothelial cells proliferation and VEGF-mediated angiogenesis

Based on evidence that the mTOR-p70S6K signalling pathway is required for VEGF stimulation of endothelial cells (Yu and Sato, 1999), we verified whether everolimus could inhibit angiogenesis at a second level, where receptor-mediated stimulation of vascular endothelial cells occurs. To investigate this hypothesis, we first evaluated the capability of everolimus to inhibit HUVECs (human umbilical vein endothelial cells) survival: HUVECs are sensitive to everolimus, with a 35% survival inhibition at 0.1 *μ*M (Figure 3D). As shown in Figure 3E, combined treatment with everolimus (0.01 *μ*M) and gefitinib (1 *μ*M) caused a 50% inhibition of HUVEC survival, with a cooperative effect compared with the single agents. We then examined the effects of everolimus on VEGF-stimulated capillary tube and network formation, and we found that this process is inhibited by everolimus 0.1 *μ*M, by gefitinib 5 *μ*M and almost suppressed by the combination of the two drugs (Figure 3F). Thus, VEGF-dependent HUVEC proliferation and tube formation seem to be very sensitive to everolimus and gefitinib inhibition.

### Combination of everolimus with gefitinib cooperatively inhibits GEO and GEO-GR colon cancer xenografts growth

**Figure 4:**
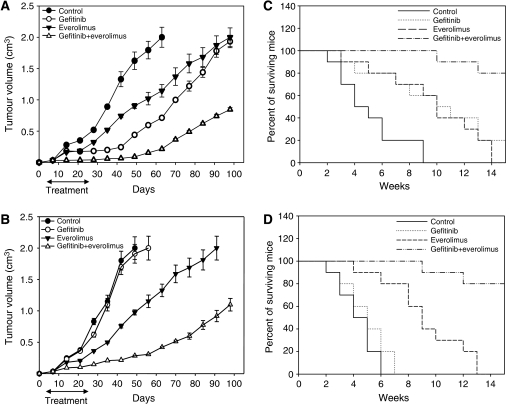
(**A** and **B**) After 7 days following tumour injection, 10 mice were treated. Tumour sizes among different treatment groups at day 56 following GEO or GEO-GR cell injection resulted statistically significant for everolimus plus gefitinib *vs* control, *vs* everolimus alone and *vs* gefitinib alone (two-sided *P*<0.0001). Bars, s.d. (**C** and **D**) Mice survival resulted statistically significant for everolimus plus gefitinib *vs* control, *vs* everolimus alone and *vs* gefitinib alone (two-sided *P*<0.0001).

To further investigate the role of everolimus on cancer cells resistant to EGFR inhibitors, we established GEO and GEO-GR xenografts by using BalbC nude mice. As expected, treatment with gefitinib alone was effective on GEO xenografts, but ineffective on GEO-GR xenografts, because, on day 56, all mice in this group reached the maximum allowed tumour size of about 2 cm^3^. Conversely, treatment with everolimus produced a 50% tumour growth inhibition, with an average tumour size of about 1.0 cm^3^ at the same time point in both xenograft models. The combination of everolimus and gefitinib caused a cooperative antitumour activity, resulting in over 90% tumour growth inhibition on day 56 (tumour size of about 0.2 cm^3^) not only in GEO, but also in GEO-GR-xenografted mice (Figure 4A and B). At the end of the experiment, 10 weeks after treatment withdrawal, GEO-GR tumour growth inhibition was still evident and tumour size was about 1 cm^3^ (Figure 4B). Comparison of tumour sizes among different treatment groups, evaluated by the Student's *t*-test, was statistically significant.

The treatment with gefitinib alone prolonged strongly the survival of GEO-xenografted mice (about 20% of mice were still alive at the end of the experiment), but no significant gain of survival was observed for GEO-GR-xenografted mice, compared with untreated animals. Mice treated with everolimus alone reached the tumour size of about 2 cm^3^ 13–14 weeks after tumour injection, with a median gain in survival compared with untreated animals of 5 weeks in GEO-xenografted mice and 7 weeks in GEO-GR mice. Combined treatment with everolimus and gefitinib produced a dramatic survival prolongation not only in GEO but also in GEO-GR-xenografted mice, in fact at the end of the experiment, 80% of mice were still alive (Figure 4C and D). Differences among the groups were calculated by log-rank test. No treatment-related side effects were observed in the treated mice.

### Combination of everolimus and gefitinib inhibits the expression of signalling proteins in GEO-GR xenografts

**Figure 5:**
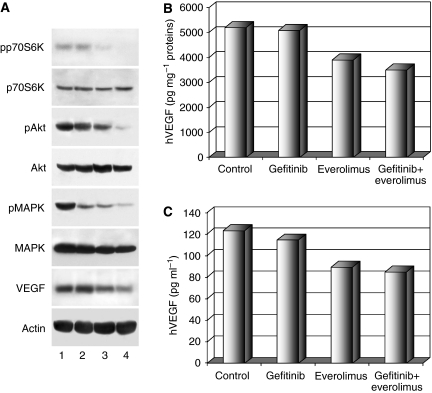
(**A**) Western blotting on total lysates from tumour specimens of two mice killed on day 25. Lane 1: untreated control; lane 2: gefitinib; lane 3: everolimus; lane 4: everolimus plus gefitinib. Bars, s.d. ELISA assays for hVEGF on total lysates from tumour specimens (**B**) and on mice serum (**C**). The results are statistically significant for everolimus plus gefitinib *vs* control and for everolimus *vs* control (two-sided *P*<0.0001), whereas they are not statistically significant for gefitinib *vs* control.

We analysed the effect of treatment on the expression of a variety of proteins having a critical role in cancer cell proliferation and angiogenesis. Western blotting analysis was performed on cell lysates from tumours removed at the end of the third week of treatment, on day 25. Everolimus markedly reduced mTOR effector phospho-p70S6K in GEO-GR xenografts, whereas gefitinib, as expected, was ineffective. The combination of everolimus with gefitinib caused a complete suppression of p70S6K phosphorylation/activation. When used alone, gefitinib and everolimus produced only a slight reduction of activity of EGFR effectors Akt and MAPK; in addition, the combined treatment efficiently reduced phopsho-Akt and phospho-MAPK expression without affecting the total amount of p70S6K, Akt and MAPK. Finally, gefitinib did not modify VEGF expression, whereas everolimus alone caused a moderate VEGF reduction further improved by the addition of gefitinib (Figure 5A).

### Combination of everolimus and gefitinib reduces the levels of hVEGF, but not of murine VEGF, in GEO-GR tumour specimens and in mice serum

To further investigate the effect of treatment on VEGF levels, we performed ELISA assays on protein extracts from tumour specimens and on serum derived from GEO-GR xenografts. Treatment with gefitinib caused only a slight reduction of both intratumour and circulating hVEGF levels, whereas everolimus treatment reduces hVEGF of about 25% both in tumour specimens and in serum. Combined treatment with everolimus and gefitinib induces a more potent inhibition of hVEGF levels as compared with treatment with single agents (Figure 5B and C). Conversely, neither single agent nor their combination affects murine VEGF (mVEGF) as compared with untreated mice (data not shown).

## DISCUSSION

In the past few years, we have learned that rational combination of targeted therapeutics may achieve a more potent antitumour effect and help to overcome the development of resistance, an emerging clinical issue often responsible for the failure of most modern antitumour approaches. In the case of EGFR and mTOR signalling pathways, many experimental data suggest that these pathways share overlapping signalling outputs (Adjei, 2006). Moreover, continued activation of PI3K/Akt signalling, which triggers mTOR, seems to contribute to the development and maintenance of an EGFR-resistant phenotype (Chakravarti *et al*, 2002; Vivanco and Sawyers, 2002; Bianco *et al*, 2003; Janmaat *et al*, 2003), and it has been found in tumour samples from cancers patients failed in EGFR-targeted therapy (Rojo *et al*, 2006). These evidences support the hypothesis that a more efficient antitumour effect may result from the combined blockade of EGFR and mTOR signalling pathways. Preclinical evidences demonstrate that this approach may be particularly effective, at least in some experimental models in which the combination of EGFR antagonists, such as gefitinib or erlotinib, and mTOR inhibitors, such as rapamycin and everolimus, resulted in an enhanced tumour cell growth control (Gemmill *et al*, 2005; Goudar *et al*, 2005; Rao *et al*, 2005; Wang *et al*, 2006; Jimeno *et al*, 2007). Recently, a pilot study including 28 heavily pretreated patients with recurrent malignant gliomas treated with the combination of gefitinib or erlotinib and rapamycin reported interesting results in terms of toxicity and tumour response (Doherty *et al*, 2006).

In the present study, we demonstrated that blocking mTOR activity might induce inhibition of cancer cell proliferation and survival. Interestingly, the antiproliferative effect exerted by the specific mTOR inhibitor everolimus does not strictly rely on the integrity of an EGFR-driven signal transduction pathway. In fact, everolimus is not only effective in tumour cells with acquired resistance to anti-EGFR drugs, but it is also able to induce a re-sensitisation against this class of inhibitors. In our experimental model, constitutive activation of the mTOR target p70S6K, which may be phosphorylated by Akt-dependent and Akt-independent mechanisms, invariably correlates with lack of response to EGFR antagonists. In addition, mTOR targeting by the specific inhibitor everolimus not only results in significant antiproliferative activity but also restores sensitivity to anti-EGFR drugs in resistant cancer cells, producing a strong reduction of Akt activation, when used in combination with anti-EGFR drugs. Although sequence has been reported to be important in some papers, in this study, we have chosen the simultaneous administration of everolimus and anti-EGFR drugs as treatment modality, because a series of experiments showed no significant differences compared with sequential administration.

It is known that inhibition of mTOR pathway correlates with decreasing of angiogenesis: in fact, rapamycin is able to reduce tumour vascularisation by promoting endothelial cell death and inducing increased susceptibility of tumour-specific vessels to thrombosis (Guba *et al*, 2005). Moreover, mTOR inhibition improves the antiangiogenic activity of anti-VEGF antibody (Stephan *et al*, 2004). It has also been clearly demonstrated that rapamycin impaires VEGF production and secretion in human cancer cells *in vitro* and endogenous VEGF in serum derived from mice bearing human tumours (Guba *et al*, 2002). These preclinical data have been confirmed by recently reported clinical data with temsirolimus in combination with antiangiogenic agents. In a phase I trial in patients with measurable stage IV clear cell renal cell carcinoma, combination therapy with temsirolimus and bevacizumab was safe and showed promising clinical antitumour activity (Merchan, 2007). A phase I study of temsirolimus in combination with sorafenib in patients with advanced solid malignancies also produced good results, without the evidence of drug–drug interactions (Patnaik, 2007). Interestingly, although EGFR inhibition induces a VEGF reduction in both protein extracts and conditioned media of wild-type tumour cells, only everolimus efficiently inhibits VEGF levels in EGFR inhibitor-resistant cells. Moreover, we have shown that the antiangiogenic effect of everolimus correlates not only with the reduction of VEGF by cancer cells but also with a direct inhibitory effect on endothelial cells, as proven by its ability to inhibit HUVEC proliferation and tubular formation alone and in combination with gefitinib.

The combined treatment with gefitinib and everolimus potentiates antitumour and antiangiogenic effects also in mice bearing GEO and GEO-GR xenografts, in which we observed a cooperative antitumour activity resulting in over 90% tumour growth inhibition on day 56, a dramatic survival prolongation; these effects correlate with a potent inhibition of Akt activation and with a serum reduction of hVEGF but not of mVEGF. The antiangiogenic activity of an EGFR and mTOR combined inhibition has been reported by others. Jimeno and co-workers (Jimeno *et al*, 2007) demonstrated that the combination of temsirolimus and erlotinib results in a synergistic antitumour effect against squamous cell carcinoma cell lines, sensitive or resistant to EGFR inhibitors.

In conclusion, mTOR inhibition causes antitumour activity in EGFR-resistant cancer cell lines and xenografts, and this effect seems to be mediated by inhibition of survival signalling pathways and angiogenesis. The combination of everolimus with an EGFR inhibitor potentiates this effect, and it may resensitise resistant cancer cells to EGFR antagonists. In light of the recent approval of mTOR inhibitors, these results support the clinical development of anti-EGFR and mTOR drugs in combination.
